# Mutational profile of *pfdhfr*, *pfdhps*, *pfmdr1*, *pfcrt* and *pfk13* genes of *P. falciparum* associated with resistance to different antimalarial drugs in Osun state, southwestern Nigeria

**DOI:** 10.1186/s41182-025-00732-6

**Published:** 2025-04-08

**Authors:** Alexandra Martín Ramírez, Akeem Abiodun Akindele, Vicenta González Mora, Luz García, Nicole Lara, Eva de la Torre-Capitán Matías, Irene Molina de la Fuente, Sulaiman Adebayo Nassar, Thuy-Huong Ta-Tang, Agustín Benito, Pedro Berzosa

**Affiliations:** 1 Centro de Investigación Biomédica en Red de Enfermedades Infecciosas, Instituto de Salud Carlos III, Madrid, Spain; 2 National Centre of Tropical Medicine, Institute of Health Carlos III, Madrid, Spain; 3 Medical Laboratory Science Department, Ladoke Akintola University of Technology, Ogbomoso, Nigeria; 4 HRH-Centre for Emerging and Re-Emerging Diseases, Ladoke Akintola University of Technology, Ogbomoso, Nigeria; 5 Complutense University of Madrid, Madrid, Spain; 6 Alcala University, Madrid, Spain

**Keywords:** Malaria, Drug-resistant *P. falciparum*, Antimalarial drug resistance, Nigeria, *pfk13*, Antimalarial drug resistance, *Pfdhps*, *Pfdhfr*, *Pfcrt*, *pfmdr1*

## Abstract

**Background:**

Nigeria accounts for the greatest burden of malaria disease globally. Malaria control requires an effective treatment after diagnosis. The efficacy of antimalarial drugs can be assessed through the analysis of genetic changes associated with reduced drug sensitivity.

**Methods:**

This study includes the analysis of the markers associated with artemisinin (*pfk13*), sulfadoxine–pyrimethamine (*pfdhfr* and *pfdhps*), and chloroquine and its derivatives (*pfmdr1* and *pfcrt*) resistances, in blood samples collected from asymptomatic children in south-western Nigeria.

**Results:**

The 25.95% of samples showed a number of mutations in *pfk13* gene. Among those, the validated, C580Y, and the candidate, R515K, mutations by WHO were detected. Twenty-seven *pfdhps* different haplotypes were observed, with the haplotype IS**G**KAA as the most prevalent (18.80%), followed by I**FG**KAA (12.78%) and I**AG**KAA (11.28%). The **VAG**K**GS** was the most common haplotype carrying the I431V mutation (10.53%). Combinations of alleles in *pfdhfr* and *pfdhps* genes provided a 40.98% of samples with the partially resistant haplotype (**IRNG**). No samples exhibited the ‘fully resistant’ or ‘super resistant’ *pfdhprf*–*pfdhps* combinations, but one sample contained mutations at *pfdhfr* 51I, 59R, and 108N with *pfdhps* 431V, 436A, A437G and 540E. The analysis of *pfcrt* 72–76 variants disclosed a 12.12% of samples with the mutant-type (CV**IET**). No double mutant *pfmdr1* haplotypes 86Y/1246Y (YY) were detected, nor was the haplotype formed by the alleles 86Y *pfmdr1* + *pfcrt* 76 T (YT).

**Conclusions:**

There was no evidence of parasite genomes harbouring multilocus mutations conferring multidrug resistance, although evidence of a validated (C580Y) and a candidate (R515K) mutation in *pfk13* gene, high frequency *pfdhfr* mutant alleles and high variability of *pfdhps* haplotypes were found in this study, which provides a baseline information essential in monitoring *P. falciparum* resistances.

**Supplementary Information:**

The online version contains supplementary material available at 10.1186/s41182-025-00732-6.

## Background

Globally, there were an estimated 263 million malaria cases in 2023 in 83 malaria endemic countries, an increase of 11 million cases compared with the previous year [1]. The WHO African Region, accounted for approximately 94% of these cases, with *Plasmodium falciparum* as the main malaria species and an estimated 597 000 malaria-related deaths in 2023 [1]. To control malaria disease, the proper management patients requires the diagnosis of *Plasmodium* parasites and the implementation of effective antimalarial drugs following diagnosis [2, 3].

The efficacy of antimalarial drugs in patient care is critical, and the failing antimalarial drugs due to parasite resistance will seriously affect patient management and recovery [2]. Antimalarial drug efficacy is monitored through therapeutic efficacy studies, which are considered the gold standard. These studies uses PCR methods to distinguish between cases with treatment failure caused by reinfection and those due to recrudescence. In addition, antimalarial drug resistance can be assessed through molecular surveillance with the molecular analysis of genetic changes associated with reduced drug sensitivity [1, 4]. Artemisinin combination therapies (ACTs) consist of an artemisinin derivative and an associated drug. The artemisinin component acts as a powerful short-acting drug that rapidly reduces parasitemia, while the associated drug acts as a second longer-acting drug that eliminate the parasites that survive artemisinin and cause recrudescence. ACTs are the first-line antimalarial therapies recommended worldwide [5]. Artemisinin-resistant *P. falciparum* is established in Southeast Asia [6], and low-level of artemisinin resistance has been identified in Africa [7]. The emergence of artemisinin partial resistance in the WHO African region is of great concern [8] due to the high malaria burden of this region. Partial artemisinin resistance is monitored through an established list of validated (associated with delayed parasite clearance in patients and in vitro*/*ex vivo confirmation) and candidate (associated with delayed parasite clearance only) *PfKelch13* markers (*pfk13)* [6, 9, 10]. Several point mutations in the *pfk13* gene have been validated to correlate with clinical artemisinin resistance (F446I, N458Y, C469Y, M476I, Y493H, R539T, I543T, P553L, R561H, P574L, C580Y, R622I and A675V) [10–12]. These non-synonymous *pfk13* mutations mainly reported in the Southeast Asia have been rarely reported in African parasites. The allele A578S allele is the most frequently observed in Africa, although it has been proven not to confer artemisinin resistance [10]. However, the R561H *pfk13* mutation has been found at high frequencies in Rwanda and confers survival at levels comparable to the C580Y mutation that dominates in the Southeast Asia, with an independent epidemiological origin from the R561H mutation and recent spreading [7]. While this mutation is associated with resistance in vitro and has been associated with slow-clearing infections in Southeast Asia, in Rwandan patients it did not lead to delayed parasite clearance. This is likely due to the high levels of acquired immunity of these patients, given that *P. falciparum* antibody titters have been strongly associated with faster parasite clearance rates in patients living in high-transmission areas like Rwanda [7, 13]. Beyond Rwanda, surveillance of *pfk13* polymorphisms has shown evidence of partial artemisinin resistance in several African countries, including Eritrea, Uganda and the United Republic of Tanzania, and it is suspected in Ethiopia, the Sudan, Namibia and Zambia [1]. The United Republic of Tanzania is the fourth country in the WHO African Region to have confirmed artemisinin partial resistance [9]. Furthermore, other countries as Democratic Republic of the Congo, have found validated *pfk13* mutations such as C469Y which decreases susceptibility to artemisinin and lumefantrine ex vivo [14, 15].

With regard to sulfadoxine–pyrimethamine (SP) resistance, they are linked to mutations in dihydrofolate reductase (*pfdhfr*) and dihydropteroate synthase (*pfdhps*) genes [2, 5]. Since SP was abandoned as a first line treatment due to the presence of resistance, the use of this combination has only been maintained for preventive treatment of vulnerable populations, such as pregnant women and children. SP is used for intermittent preventive treatment (IPT) of malaria in infants (IPTi), during pregnancy (IPTp), and in combination with amodiaquine (AQ) in children for the Seasonal Malaria Chemoprevention (SMC) [2, 16]. The use of IPT with SP is strongly recommended in areas of moderate to high *P. falciparum* malaria transmission; in fact, it is now recommended for all pregnant women, regardless of the number of pregnancies [8, 9]. Studies indicate that community use of SP, whether for treatment or chemoprevention, is often followed by increases in community prevalence of resistance mutations in both *pfdhfr* and *pfdhps* [17–19]. Mutations in *pfdhfr* and *pfdhps P. falciparum* genes are associated with decreased parasite sensitivity to the antifolate drugs [2]. Mutations on *pfdhfr* gene*,* C50R, N51I, C59R, S108N and I164L, confer pyrimethamine resistance [2, 20], while S436A, A437G, K540E, A581G and A613S/T *pfdhps* mutations confer sulfadoxine resistance [2, 20, 21]. The *pfdhfr* C**IRN**I triple mutant haplotype, which confers a high level of pyrimethamine resistance, has been spreading in Africa since the 1980s. When combined with the I164L mutation, it results in the highest grade pyrimethamine resistance and leads to the rapid spread of antifolate resistance [22]. The A437G *pfdhps* mutation, alone or in combination with K540E, is responsible for sulfadoxine–pyrimethamine treatment failures [23]. The I431V *pfdhps* mutation, first described in Nigeria in 2007 [24], has been found with high frequency in northwestern African countries, including Nigeria [23]. Notably, the geographical distribution of I431V mutation is the opposite to K540E and they have never been observed together [23]. Three combinations of *pfdhfr* plus *pfdhps* single nucleotide polymorphisms (SNPs) related to SP resistance have been described: partially resistant (quadruple mutant: *pfdhfr* 51I/59R/108N + *pfdhps* 437G, alone or in combination with 436A), **IRNG** haplotype; fully resistant (quintuple mutant: *pfdhfr* 51I/59R/108N + *pfdhps* 437G/540E), **IRNGE** haplotype; and, super resistant (sextuple mutant: *pfdhfr* 51I/59R/108N + *pfdhps* 437G/540E/581G and/or *pfdhfr* 164L), **IRNGEG** haplotype [21, 23, 25]. Super resistant haplotypes have been reported in areas, where IPTi and IPTp have failed [25].

Infections that fail chloroquine (CQ) or its derivatives are related to mutations in the CQ resistance transporter (*pfcrt*) gene and on multidrug resistance gene (*pfmdr1*) [26]. The mutation of *P. falciparum pfcrt* K76T has been confirmed to be closely related to CQ resistance [27, 28], and is often accompanied by M74I and N75E mutations [29]. Piperaquine, an antimalarial drug often combined with dihydroartemisinin, has also shown therapeutic failures, with *pfcrt* as the major contributor [30]. Furthermore, mutations in *pfmdr1*, such as N86Y, S1034C, Y184F, N1042D and D1246Y, are involved on resistance to multiple drugs, such as CQ, quinine, AQ, lumefantrine and piperaquine [26, 31]. Studies on *pfmdr1* gene have identified that the amino-terminal mutations (N86Y and Y184F) are more common to Asian and African parasites, whereas the three carboxy-terminal mutations (S1034C, N1042D and D1246Y) are found more often in South American isolates [32]. The N86Y *pfmdr1* mutation contributes to resistance to CQ and AQ, while sensitize parasites to lumefantrine, mefloquine and dihydroartemisinin. On the other hand, the Y184F mutation alone has a limited impact [32]. Haplotypes of *pfmdr1* encoding Tyr at codons 86, 184 and 1246, YYY haplotype, are associated with AQ resistance [33]. In addition, the presence of both mutants *pfcrt* K76T and *pfmdr1* N86Y alleles in parasites is associated with parasite response to CQ [27, 32] and in vivo AQ resistance [31]. An increased *pfmdr1* gene copy number is also linked to resistance to mefloquine and artesunate [34].

Nigeria, located in the western part of the African mainland, is one of four countries that accounts for nearly half of global malaria cases, with a malaria burden of 26% in the last World Malaria Report [1] and an increase in recent years due to COVID-19 pandemic and other factors [9]. *P. falciparum* is the major pathogen causing malaria in Nigeria (94–98% of malaria cases), with less frequently infections by *P. malariae, P. ovale* and *P. vivax* [35]. In southwest Nigeria, malaria is highly endemic and transmission occurs throughout the year [36], but mainly during the rainy season (April to October) [37]. ACTs were introduced in Nigeria in 2005, with artemether–lumefantrine (AL) as first-line treatment and artesunate–amodiaquine as the alternative [38]. Although Nigeria is not among the WHO African countries that have shown the emergence of resistance associated with ACTs [9, 14], high levels of resistance have been found in other resistance markers as *pfdhfr, pfdhps* and *pfmdr1* [2, 21]. Due to the threat that antimalarial drug resistance poses to malaria control and the dynamic epidemiology, the information on the pattern of evolution of genes conferring resistance to different antimalarial drugs, to ensure that the most effective treatments are selected in the national treatment policy, is essential. This study aimed to determine the prevalence of multi-locus anti-malarial resistance markers in *P. falciparum* isolates collected in Osun State, southwestern Nigeria**,** in 2021. This includes analysis of markers associated with artemisinin resistance (*pfk13*), SP resistance (*pfdhfr and pfdhps*), and CQ and its derivatives resistance (*pfmdr1* and *pfcrt*).

## Methods

### Study design and sample collection

**Fig. 1:**
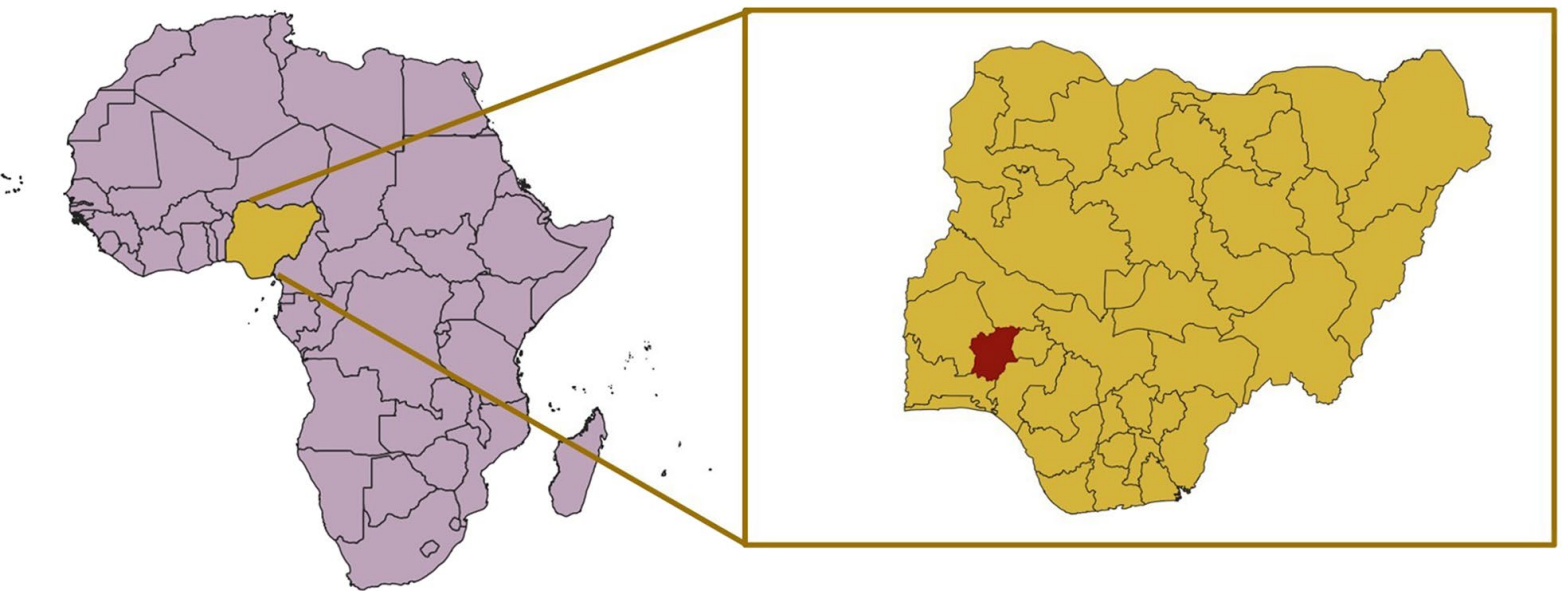
Map showing the location of Nigeria in Africa. Osun state, where the sampling took place, is highlighted in red

Samples from asymptomatic children in Ore, a rural community in Osun State, southwestern Nigeria (Fig. 1), were collected from February to May 2021, after obtaining the corresponding written informed consent from their parents/caregivers. Blood samples were spotted on Whatman 903^™^ paper (GE Healthcare Bio-Sciences Corp.), stored dry and individually inserted in a zip-lock bag with silica gel. All dried blood spots (DBSs) were stored at − 20 °C until their use. The samples were transferred to the National Centre of Tropical Medicine, Institute of Health Carlos III, Madrid (Spain), for molecular analysis. Malaria diagnosis was made in field by thick blood smear microscopy and rapid diagnostic tests (RDTs). Demographic data, such as sex and age, were recorded for each sample.

Samples diagnosed as *P. falciparum* by Nested Multiplex Malaria–PCR (NM–PCR) [39, 40] were selected to screen resistance markers *pfdhfr, pfdhps*, *pfmdr1*, *pfcrt* and *pfk13.*

### Ethics approval

Approval to conduct this study was obtained from the Research and Ethics Committee of Osun State, Ministry of Health, Osogbo, Nigeria (reference number: OSHREC/PRS/569 T/131). All parents/caregivers from children gave written informed consent to participate using the template approved by the Ethics Committee before the study. Permission was sought from the local inspector of education, head teacher and community leaders.

### DNA extraction

DNA was extracted from the DBSs samples using the saponin–chelex method with a 5 mm diameter-filter paper disc [41, 42]. The extracted DNA was immediately used for PCR reactions and the remains were stored at − 20 °C.

### Nested multiplex malaria–PCR (NM–PCR)

NM–PCR was used as the reference method, and it was performed on all samples to characterize them. This method is accredited by UNE–EN ISO 15189:2022 (N: 175/LE1213) and was performed according to the manufacturer’s and original authors’ recommendations [39, 40]. The first PCR reaction amplifies *Plasmodium* spp. and includes a human internal amplification control, while the second reaction uses the amplified DNA of the first PCR reaction to identify the infecting species of *P. vivax, P. falciparum, P. ovale* and *P. malariae* by the corresponding size of the amplified fragments.

### Molecular markers for resistance nested-PCR

The *P. falciparum* genes *pfk13, pfdhfr*, *pfdhps*, *pfcrt* and *pfmdr1* were analysed. Nested-PCR for *pfk13* gen protocol was described by Ariey et al*.* [43, 44] with some modifications. Briefly, Polymerase Hot-Start (Biotools B&M Labs, S.A., Spain) at a final concentration of 0.028 U/mL was used. For the first PCR, 5 µL of isolated genomic DNA were used, along with 0.25 µM of each primer. The final volume of PCR reaction was 25 µL. The second PCR was performed with 0.25 µM of each primer, with a final volume of 50 µL. Conditions for first *pfk13* PCR consisted of denaturation at 95 °C for 6 min, followed by 30 cycles at 94 °C for 30 s, 58 °C for 2 min, and 72 °C for 2 min. The final cycle was followed by an extension time at 72 °C for 10 min. Second *pfk13* PCR consisted of an initial denaturation for 6 min at 94 °C, followed by 40 cycles at 94 °C for 30 s, 60 °C for 1 min, and 72 °C for 1 min. The final extension time was 10 min at 72 °C.

Nested-PCR mutation screening protocols for *pfdhfr*, *pfdhps*, *pfcrt* and *pfmdr1* genes were performed as described by Plowe et al. with minor modifications [20]. Two microliters of DNA were added for the first reaction and 2 µL of first PCR were added to the second reaction in Nested-PCR *pfdhfr*, *pfdhps*, *pfcrt* and *pfmdr1.* Conditions of *pfdhfr, pfdhps* and *pfcrt* first PCR consisted of denaturation at 95 °C for 5 min, followed by 45 cycles at 92 °C for 30 s, 45 °C for 30 s, and 65 °C for 45 s. The final cycle was followed by an extension time at 72 °C for 15 min. Second *pfdhfr, pfdhps* and *pfcrt* PCR consisted of denaturation at 95 °C for 5 min and 25 cycles at the same temperatures than first PCR but 30 s for the extension step. Conditions of first and second *pfmdr1* PCR were a denaturation at 95 °C for 3 min, followed by 35 cycles at 93 °C for 30 s, 52 °C for 30 s, and 72 °C for 45 s. The final cycle was followed by an extension time at 72 °C for 5 min.

PCR products were separated by electrophoresis on a 2% agarose gel, stained with Condasafe stain (Condalab, Spain) and identified based on the amplified fragment size visualized under an ultraviolet transilluminator (BioRad chemiDoc XRS +; BioRad). The PCR products were purified using Ilustra exoprostar one-step (GE Healthcare Life Sciences) in accordance with the manufacturer’s instructions. Samples were sequenced from both directions using the forward and reverse primers of the second PCR at a final concentration of 6 pmol/µL, using a standard dye terminator (Big Dye Terminator v3.1 Cycle Sequencing kit) in an ABI PRISM 3730 XL Analyser. Sequences were compared with Genebank database using BLAST (Basic Local Alignment Search Tool) [45].

To identify mutations in analysed resistance markers, the *P. falciparum* 3D7 clone sequence was used as the reference for comparison with all sequences obtained from each sample using BioEdit 7.2 software.

All polymorphisms were analysed in *pfk1*3 gene, while specific codon positions were examined for the rest of the genes: 51, 59, 108 and 164 in the *pfdhfr* gene; 431, 436, 437, 540, 581 and 613 in the *pfdhps* gene; 72, 73, 74, 75 and 76 in the *pfcrt* gene; and 86, 130, 184, 1034, 1042, 1109 and 1246 in the *pfmdr1* gene. Variants were translated and haplotypes inferred.

### Data analysis

Polymorphism analysis was conducted through the examination of the obtained sequences using BioEdit 7.2 software. The mutations and haplotypes frequencies were calculated for each gene. Mutations and haplotype frequencies were calculated as the ratio of the sample(s) with the mutation to the total number of individual samples genotyped successfully.

## Results

A total of 350 samples were collected in the study. Mean age was 10.5 ± 3.60 years and 156 samples (44.57%) belonged to female children.

Microscopy and Rapid Diagnostic Tests (RDTs) were positive in 93.71% (328/350) and 79.43% (278/350) of the samples, respectively. Geometric mean parasite density was 1834 parasites/µL (range 40–5360 parasites/µL) in microscopy positive samples. NM–PCR provided positive results for 89.43% (313/350) samples. All positive samples were identified as *P. falciparum*, and no mixed infections were detected.

### *Pfk13* resistance marker results

**Fig. 2:**
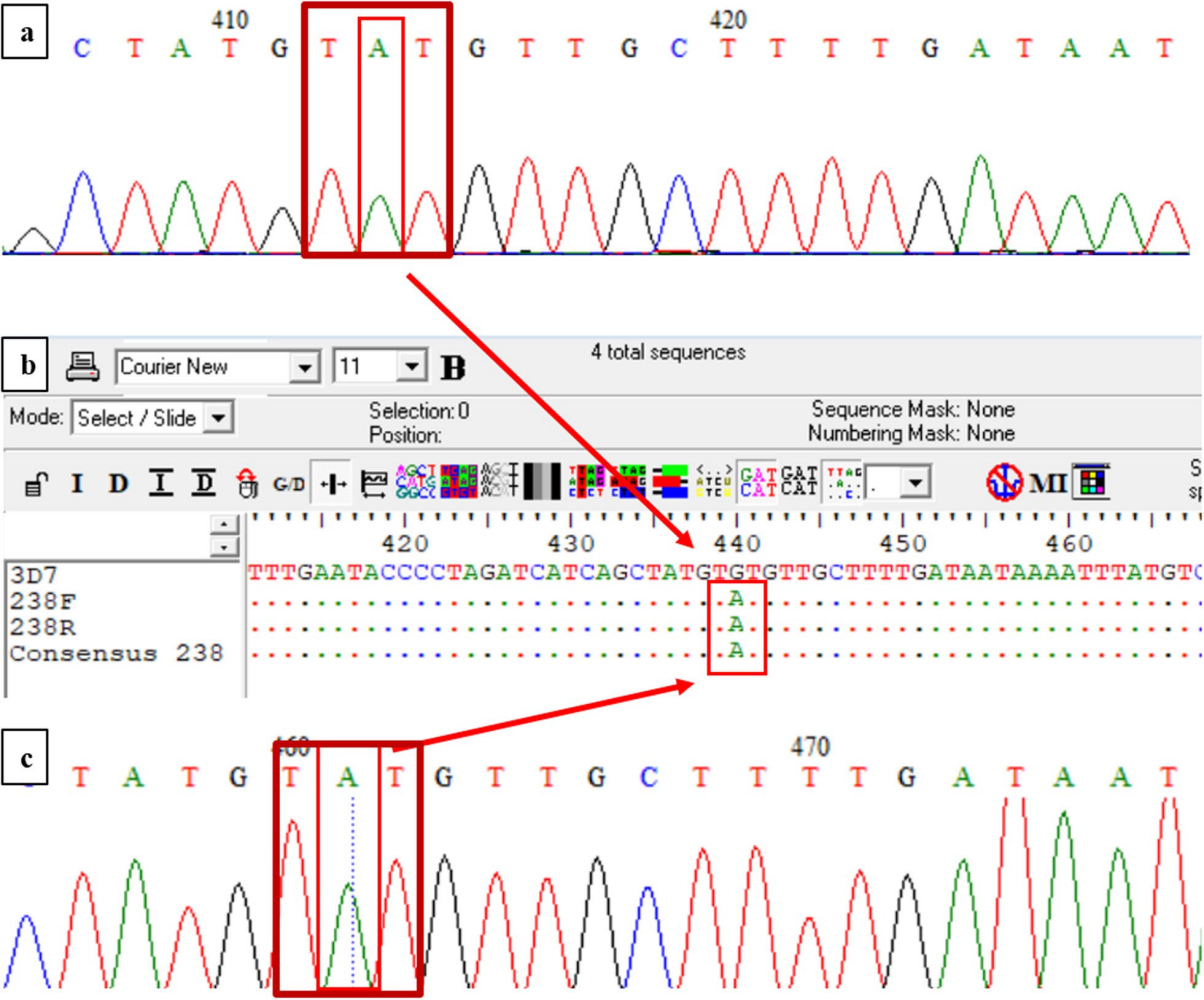
Validate marker C580Y detected in one sample. Electropherogram of the forward (**a**) and reverse complement (**c**) sequences with BioEdit 7.2 software. Alignment of the forward, reverse complement and consensus sequences compared to the reference *P. falciparum* 3D7 sequence (**b**). The sequence alignment images (**b**) displays the TGT triplet (wild type) in the reference sequence, while the forward, reverse complement and consensus sequences shows the substitution of G with A. Nucleotides corresponding to the TAT (mutant codon) are highlighted with boxes

**Fig. 3:**
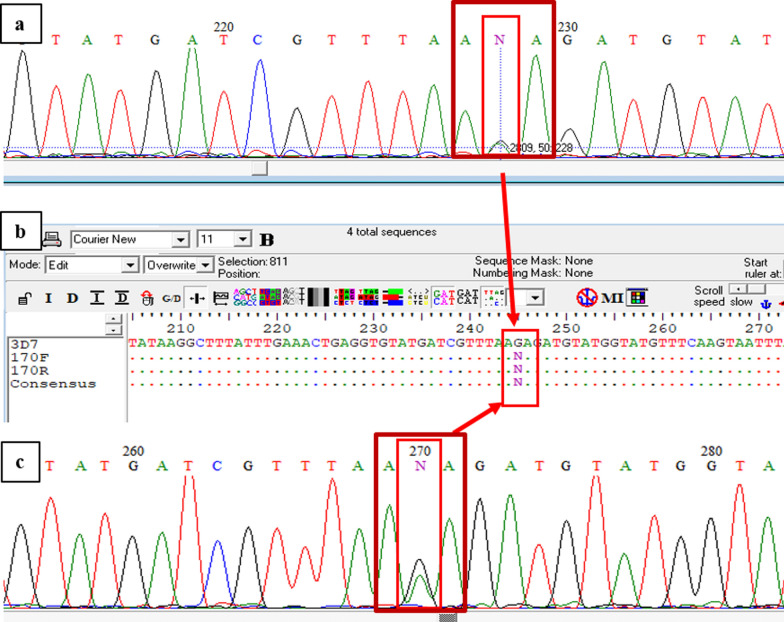
Candidate marker R515K. Electropherogram of the forward (**a**) and reverse complement (**c**) sequences with BioEdit 7.2 software. Alignment of the forward, reverse complement and consensus sequences compared to the reference *P. falciparum* 3D7 sequence (**b**), where the N nucleotide (highlighted with boxes) corresponds to the mixed peak with the mutant (A) and the wild-type (G) nucleotides, with almost the same intensity. The sequence alignment images (**b**) displays the AGA triplet (wild type) in the reference sequence, while the forward, reverse complement and consensus sequences shows the substitution of G with the mix A/G

The *pfk13* gene was amplified and sequenced in 262 out of 313 *P. falciparum–*NM–PCR positive samples, while 51 samples failed to amplify by *pfk13–*PCR after several attempts with varying PCR conditions. The majority of the *pfk13* genes studied (194/262, 74.05%) were wild type. The frequency of parasites with *pfk13* mutations was 25.95% (68/262). Most samples contained a single SNP, while five samples contained two SNPs, two samples contained three SNPs and one sample contained five SNPs. Of the 65 polymorphism detected, sixteen were synonymous, and 49 were non-synonymous (Table 1). The WHO validated mutation, C580Y, was found in one sample (Fig. 2). In addition, one sample had a candidate mutation mixed with wild-type allele, in position 515 (Fig. 3). The non-synonymous G449D, N458D, P527L, P553S/Q and synonymous C469C polymorphisms were found in the same position as validated or candidate markers (G449A, N458Y, P527H, P553L and C469Y/F, respectively). In addition, non-synonymous V494I, D516N, S536P, C542R, A569T and S623N and synonymous P475P mutations were contiguous to Y493H, R515K, N537I/D, I543T, V568G, R622I and M476I validated or candidate markers, respectively.

**Table 1:** SNPs and their frequencies detected in the *pfk13* gene after amplification and sequencing

Codon	Sequence wild type (nt)	AA wild type	Sequence mutant allele (nt)	AA mutant	Mutation	Number (n)	Frequency (%)
434	ttt	F	**a**tt	I	F434I	5	6.17%
443	cca	P	cc**c**	P	P443P	1	1.23%
447	tgt	C	**ga**t	D	C447D	1	1.23%
447	tgt	C	t**a**t	Y	C447Y	1	1.23%
449	ggt	G	g**a**t	D	G449D	1	1.23%
451	ttt	F	tt**g**	L	F451L	1	1.23%
458	aat	N	**g**at	D	N458D	1	1.23%
461	gaa	E	**a**aa	K	E461K	1	1.23%
469	tgc	C	tg**t**	C	C469C	1	1.23%
475	cct	P	ccc	P	P475P	1	1.23%
478	acc	T	**g**cc	A	T478A	2	2.47%
478	acc	T	acg	T	T478T	1	1.23%
486	gct	A	g**g**t	G	A486G	1	1.23%
494	gtt	V	**a**tt	I	V494I	1	1.23%
499	aac	N	aa**t**	N	N499N	1	1.23%
507	gaa	E	**a**aa	K	E507K	1	1.23%
508	act	T	a**g**t	S	T508S	1	1.23%
509	gag	E	ga**a**	E	E509E	1	1.23%
512	gat	D	**a**at	N	D512N	1	1.23%
513	cgt	R	**t**gt	C	R513C	1	1.23%
515	aga	R	a**a**a	K	R515K	1	1.23%
516	gat	D	**a**at	N	D516N	1	1.23%
527	cct	P	c**t**t	L	P527L	1	1.23%
535	acg	T	ac**t**	T	T535T	1	1.23%
536	tca	S	**c**ca	P	S536P	1	1.23%
542	tgt	C	**c**gt	R	C542R	1	1.23%
546	tat	Y	ta**c**	Y	Y546Y	1	1.23%
548	ggc	G	g**c**c	A	G548A	1	1.23%
553	ccg	P	c**a**g	Q	P553Q	1	1.23%
553	ccg	P	**t**cg	S	P553S	1	1.23%
556	gaa	E	**a**aa	K	E556K	3	3.70%
557	gca	A	gc**g**	A	A557A	1	1.23%
557	gca	A	**a**ca	T	A557T	1	1.23%
564	gca	A	**a**ca	T	A564T	1	1.23%
569	gca	A	**a**ca	T	A569T	1	1.23%
573	acc	T	**g**cc	A	T573A	1	1.23%
578	gct	A	**a**ct	T	A578T	1	1.23%
578	gct	A	**t**ct	S	A578S	5	6.17%
580	tgt	C	t**a**t	Y	C580Y	1	1.23%
589	gtc	V	gt**g**	V	V589V	1	1.23%
595	ggt	G	g**a**t	D	G595D	1	1.23%
613	caa	Q	ca**t**	H	Q613H	2	2.47%
614	ttt	F	**c**tt	L	F614L	1	1.23%
623	agt	S	a**a**t	N	S623N	1	1.23%
626	gca	A	**a**ca	T	A626T	1	1.23%
627	gct	A	**a**ct	T	A627T	1	1.23%
630	tac	Y	ta**t**	Y	Y630Y	2	2.47%
638	gga	G	gg**g**	G	G638G	1	1.23%
646	ata	I	at**g**	M	I646M	1	1.23%
652	caa	Q	**t**aa	END	Q652END	1	1.23%
653	tat	Y	t**t**t	F	Y653F	1	1.23%
655	cca	P	**t**ca	S	P655S	1	1.23%
657	aat	N	a**g**t	S	N657S	1	1.23%
661	caa	Q	c**c**a	P	Q661P	1	1.23%
668	gag	E	**a**ag	K	E668K	1	1.23%
668	gag	E	**c**ag	Q	E668Q	1	1.23%
668	gag	E	ga**a**	E	E668E	1	1.23%
677	aca	T	a**t**a	I	T677I	1	1.23%
687	gga	G	gg**c**	G	G687G	1	1.23%
688	gaa	E	**c**aa	Q	E688Q	1	1.23%
690	ggc	G	gg**t**	G	G690G	1	1.23%
692	gtt	V	**a**tt	I	V692I	4	4.94%
692	gtt	V	**c**tt	L	V692L	1	1.23%
699	ttt	F	tt**c**	F	F699F	1	1.23%
702	gat	D	**a**a**a**	K	D702K	1	1.23%

### *Pfdhfr* and *pfdhps* resistance markers results

Successful genotyping for both *pfdhfr* and *pfdhps* genes was obtained in 266 samples out of 313.

**Fig. 4:**
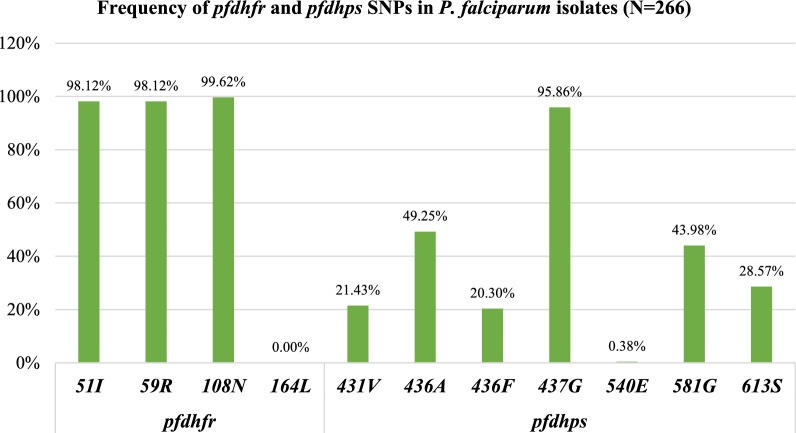
Prevalence of single point mutations in the *pfdhfr* and *pfdhps* genes of the *P. falciparum* isolates (N = 266)

Frequency of 51I, 59R and 108N *pfdhfr* mutant alleles was 98.12%, 98.12% and 99.62%, respectively, with no detection of 164L *pfdhfr* mutation (Fig. 4). 96.99% (258/266) of the samples showed the triple *pfdhfr* mutant (**IRN**I) haplotype (Table 2).

**Table 2:** Prevalence and percentage of combined *pfdhfr* and *pfdhps* haplotypes detected in *P. falciparum* isolates (*N* = 266)

Combination of mutated codons	Haplotypes	Number (n)	Percentage (%)
51/59/108/437 (partially resistant) [25]	**IRN**I + IS**G**KAA	47	17.67
51/59/108/437/540 (fully resistant) [25]	**IRN**I + IS**GE**AA	0	0.00
51/59/108/437/540/581 (super resistant) [25]	**IRN**I + IS**GEG**A	0	0.00
51/59/108/436/437	**IRN**I + I**AG**KAA	30	11.28
51/59/108/436/437	**IRN**I + I**FG**KAA	32	12.03
51/59/108/431/436/437/540	**IRN**I + **VAGE**AA	1	0.38
51/59/108/437/613	**IRN**I + IS**G**KAs	2	0.75
51/59/108/437/581/613	**IRN**I + IS**G**KGS	7	2.63
51/59/108/437/581	**IRN**I + IS**G**K**G**A	21	7.89
51/59/108/431/436/437	**IRN**I + **VAG**KAA	6	2.26
51/59/108/431/436/437/581/613	**IRN**I + **VAG**K**GS**	26	9.77
51/59/108/431/436/437/581	**IRN**I + **VAG**K**G**A	12	4.51
51/59/108/431/437/613	**IRN**I + **V**S**G**KA**S**	1	0.38
51/59/108/431/437/581/613	**IRN**I + **V**S**G**K**GS**	1	0.38
51/59/108/431/437/581	**IRN**I + **V**S**G**K**G**A	3	1.13
51/59/108/436/581	**IRN**I + I**A**AK**G**A	3	1.13
51/59/108/436/581/613	**IRN**I + I**A**AK**GS**	1	0.38
51/59/108/436	**IRN**I + I**A**AKAA	3	1.13
51/59/108/436	**IRN**I + I**F**AKAA	1	0.38
108/436/437	NC**N**I + I**FG**KAA	1	0.38
59/108/436/437	N**RN**I + I**FG**KAA	1	0.38
51/59/108/436/613	**IRN**I + I**A**AKA**S**	1	0.38
51/59/108/436/437/613	**IRN**I + I**AG**KA**S**	16	6.02
51/59/108/436/437/581	**IRN**I + I**AG**K**G**A	15	5.64
51/59/108/436/437/581	**IRN**I + I**FG**K**G**A	11	4.14
51/59/108/436/437/581/613	**IRN**I + I**AG**K**GS**	11	4.14
59/108/436/437/581/613	N**RN**I + I**AG**K**GS**	1	0.38
51/108/437	**I**C**N**I + IS**G**KAA	2	0.75
437	NCSI + IS**G**KAA	1	0.38
51/59/108/431/436/581/613	**IRN**I + **VF**AK**GS**	1	0.38
51/59/108/436/437/613	**IRN**I + I**FG**KA**S**	1	0.38
51/59/108/436/437/581/613	**IRN**I + I**FG**K**GS**	1	0.38
51/59/108/431/436/437/613	**IRN**I + **VAG**KA**S**	3	1.13
51/108/431/436/437/581/613	**I**C**N**I + **VAG**K**GS**	1	0.38
59/108/431/436/437/581/613	N**RN**I + **VAG**K**GS**	1	0.38
51/59/108/431/581/613	**IRN**I + **V**SAK**GS**	1	0.38

The evaluation of the prevalence of SNPs *pfdhps* alleles showed the 437G as the most frequent mutation (95.86%), followed by 436A (49.25%) and 581G (43.98%) mutations (Fig. 4)*.* Fifty-seven samples (21.43%) showed 431 V mutation, and just one sample showed the 540E *pfdhps* SNP (Fig. 4).

The combination of mutations at codons 431, 436, 437, 540, 581 and 613 of *pfdhps* genes provided 27 different *pfdhps* haplotypes (Supplementary Table 1). The A437G single mutant (IS**G**KAA) was the most prevalent haplotype (50/266; 18.80%), followed by I**FG**KAA (12.78%) and I**AG**KAA (11.28%) haplotypes, while the **VAG**K**GS** quintuple mutant was the most common haplotype carrying the I431V mutation (10.53%). The only sample with the 540E mutation was the *pfdhps* haplotype **VAGE**AA (Supplementary Table 1).

Combinations of SNPs in both *pfdhfr* and *pfdhps* genes (Table 2) were classified according to the three haplotypes, named ‘partially resistant’, ‘fully resistant’ and ‘super resistant,’ as described by Naidoo [25]. The frequency of the ‘partially resistant’ haplotype (**IRNG** haplotype, including the triple **IRN**I *pfdhfr* and mutation at codon 437 *pfdhps* alone) was 17.67%. However, when combined with 436A/F mutation, the frequency increased to 40.98%. No ‘fully resistant’ (**IRNGE** haplotype, including the triple **IRN**I *pfdhfr* and mutation at codon 437G *pfdhps*, plus 540E) or super resistant (**IRNGEG** haplotypes) haplotypes were detected, but one sample exhibited the **IRN**I *pfdhfr* combined with mutations at codons 436A plus 437G plus 540E *pfdhps* haplotype (Table 2).

### *Pfcrt* and *pfmdr1* resistance markers results

**Fig. 5:**
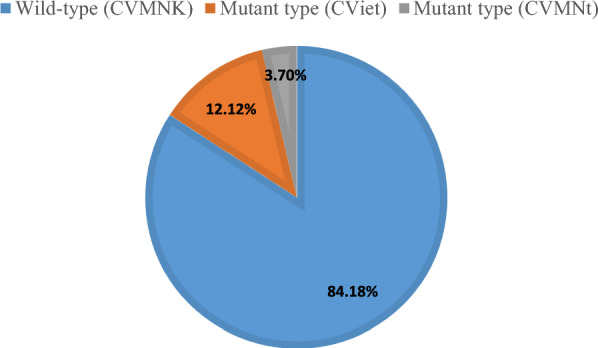
Prevalence of haplotypes at codons 72–76 of the chloroquine resistance transporter gene (*pfcrt*). Wild-type haplotype CVMNK = cysteine–valine–methionine–asparagine–lysine, and mutant-type haplotypes CV**IET** = cysteine–valine–isoleucine–glutamic acid–threonine and CVMN**T** = cysteine–valine–methionine–asparagine–threonine

A total of 297 out of 313 PCR positive samples were successfully amplified and sequenced for polymorphisms in the *pfcrt* gene covering codons at 72, 73, 74, 75 and 76 positions. The mutant K76T allele was found in 15.82% of samples (Table 3). The wild-type CVMNK haplotype was detected in 250 out of 297 samples (84.18%), while mutant-type (CVIET) was less prevalent (36/297, 12.12%) (Fig. 5). Nine out of 36 samples with the mutant-type haplotype (CVIET) had mixed mutant and wild-type haplotypes. A small proportion of samples (11/297, 3.70%) showed a haplotype with only the K76T allele (CVMNT). No SNPs were detected at 72 and 73 positions of *pfcrt* gene.

**Table 3:** Frequencies of SNPs and haplotypes in *pfcrt* and *pfmdr1 P. falciparum* samples

	Codon mutation	No. of isolates (*n*)	Frequency (%)
Genes			
*pfcrt* (*n* = 297)	M74I	36	12.12
	N75E	36	12.12
	K76T	47	15.82
*pfmdr1* (*n* = 262)	N86Y	7	2.67
	E130K	0	0.00
	Y184F	208	79.39
	S1034C	0	0.00
	N1042D	2	0.76
	V1109I	0	0.00
	D1246Y	3	1.15

*Pfmdr1* resistance markers were analysed in 262 samples. The 86Y mutant allele was detected in 7 out of 262 samples (2.67%), while 1246Y mutant allele was present in 1.15% (3/262) (Table 3). The linked polymorphism *pfmdr1* 184F was present at 79.39% frequency, whereas the double mutant *pfmdr1* haplotype 86Y/1246Y (YY) was not detected. The described SNPs 130 K, 1034C and 1109I were not detected in any sample. SNPs 86D and 1042I were found in three and two samples, respectively.

Combined mutations on *pfcrt* and *pfmdr1* were successfully determined in 253 samples. No samples were found with the double mutant haplotype formed by the mutations in 86Y *pfmdr1* plus 76 T *pfcrt* (YT).

## Discussion

The aim of this study was to determine the frequency of *pfk13, pfdhfr, pfdhps, pfcrt* and *pfmdr1 *genetic polymorphisms. The ability of malaria parasites to develop resistance to antimalarial drugs underscores the importance of using of molecular markers for drug-resistant surveillance in malaria control programs. Identifying those mutations is crucial for making proper policy adjustments [46].

In Nigeria, ACTs were introduced in 2005 and, to date, resistance has not been confirmed [9, 46]. This study showed a broad array of rare non-synonymous mutations in *pfk13* gene that were not validate or candidate markers by WHO. Most polymorphisms (58/65) were observed only once. The synonymous C469C polymorphism has been detected in other African countries, such as Mozambique, Senegal, Ghana and Equatorial Guinea [4, 10, 20, 47], while G690G and E509E has previously been reported in Mozambique and Kenya, respectively [4, 10]. The A578S mutation, the most frequently observed African allele, was found in this study with a frequency of 6.17%. However, no clinical or in vitro artemisinin resistance has been associated with this mutation [11]. Unlike previous studies [10, 46, 48–52], which found no validated mutations associated with artemisinin resistance, one validate (C580Y) and one candidate (R515K) marker were found in this study. To our knowledge, this is the first evidence of detection of validated marker C580Y in Nigeria. A previous study in Nigeria [53] reported two suspected samples with C580Y mixed alleles (wild type and mutant) using a specific real-time PCR assay; however, Sanger sequencing against the reference strain *P. falciparum* 3D7 failed to confirm the mutant type allele. The presence of C580Y can decrease the binding capacity to phosphatidylinositol 3-kinase (PI3K), which contributes to artemisinin resistance in vitro [54, 55]. This finding may raise concerns about artemisinin resistance in Nigeria, the country with the highest number of malaria cases and deaths among malaria endemic nations [9]. In addition, 13 mutations were detected in the same or adjacent position to validate or candidate *pfk13* markers. The polymorphisms need to be carefully monitored and assessed in future studies, as they indicate instability in these genomic regions, which could potentially affect antimalarial activity of artemisinins [56, 57].

The wide range of parasites with non-candidate or validated mutations detected in this study highlights the high genetic variability of *P. falciparum* parasites circulating in Nigeria. The role of these mutations in artemisinin sensitivity/resistance is difficult to elucidate, as no clinical study or in vitro tests were conducted here. Thus, it is imperative that in vivo and in vitro studies be conducted to validate the potential role of these mutations in ACT resistance [48].

Regarding to sulfadoxine–pyrimethamine (SP) resistance, this is determined by mutations in *pfdhps* and *pfdhfr* genes of *P. falciparum*. The WHO recommends SP as part of a package of interventions for malaria prevention and control during pregnancy, as the primary antimalarial medicine with efficacy and safety for Intermittent Preventive Treatment (IPT) in areas of moderate or high transmission of *P. falciparum* [58]*.* Children also benefit from SP-based prevention strategies, such as Seasonal Malaria Chemoprevention (SMC), where SP is combined with AQ, originally recommended for children under 6 years of age, but now extended to other children in some locations and to countries with highly seasonal variation in malaria burden [59]; and Perennial Malarial Chemoprevention (PMC), formerly known as Intermittent Preventive Treatment in infants (IPTi), that includes children beyond their first or second year of life [59]. The presence of SP resistance significantly impacts on IPTp, IPTi, and SMC strategies, and severely influences the achievement of malaria prevention in African countries [22]. SP-based prevention strategies are recommended based on 540E *pfdhps* mutations prevalence. Accordingly, the Nigeria’s National Malaria Strategic Plan 2021–2025 includes SMC for eligible children as well as a pilot study on IPTi for infants under 12 moths [60]. Our results on SP resistance markers, *pfdhps* and *pfdhfr* genes, showed the triple mutation 51I, 59R and 108N in the *pfdhfr* gene almost fixed, whereas 164L was absent. The 164L mutation, often co-occurring with the triple mutation 51I, 59R and 108N, is associated with high levels of SP resistance and is common in Asia and South America regions, while it has not spread in sub-Saharan Africa, allowing SP to retain some efficacy in preventing malaria during pregnancy [61]. The *pfdhps* locus displayed considerable variability, generating 27 distinct haplotypes. The prevalence of *pfdhps* mutations was high, with 437G, 436A and 581G being the most prevalent mutations, while the 540E mutation was almost absent, detected in only one sample. The low prevalence of 540E mutation (0.38%), which is rarely found in West and Central Africa [52], supports the continued use of SP in PMC, that according to the WHO guidelines remains effective, where the 540E mutation prevalence is ≤ 50% [16].

The combination of *pfdhfr* and *pfdhps* haplotypes showed a high percentage of samples (40.98%) with the ‘partially resistant’ combination of *pfdhfr* N51I, N59R, and S108N with *pfdhps* A437G ± S436A/F. No samples exhibited the ‘fully resistant’ or ‘super resistant’ *pfdhfr*–*pfdhps* combinations described by Naidoo, but one sample contained mutations at *pfdhfr* 51I, 59R, and 108N with *pfdhps* 431V, 436A, A437G and 540E. The absence of samples with fully and super resistant *pfdhfr*–*pfdhps* combinations, suggests that SP remains effective for prevention strategies in the study area. These findings align with the known geographical distribution of resistance haplotypes, where the ‘partially resistant’ haplotype is predominant in West Africa, whereas the ‘fully resistant’ and ‘super resistant’ combinations are more frequent in East Africa [25, 62]. However, the quintuple mutant haplotype (**VAG**K**GS**) emerging in Nigeria and Cameroon [33], has been found to be 10.53% in this study, a lower prevalence than other studies conducted in Nigeria [63]. The impact of this haplotype on SP efficacy remains uncertain, but its increasing prevalence over the years seems to indicate that it confers selective advantage in the presence of SP drug pressure, displacing more sensitive haplotypes [64]. This trend raises significant concern for the future efficacy of SP-based prevention strategies.

Mutations at codons 72, 73, 74, 75 and specially 76 of *pfcrt* cause resistance to CQ and its derivatives [27, 28, 61]. In 2005, Nigeria changed its malaria treatment policy from CQ to ACT treatments, after efficacy studies indicated that chloroquine and SP were no longer adequate for national first line use [38]. Since then, several studies in Nigeria [50, 53] and other African countries [4, 20, 32, 65, 66] have showed a reduction in the detection of these mutant alleles compared to wild-type haplotypes. However, some studies conducted in Nigeria have documented high prevalence of CV**IET** haplotype despite withdrawal of CQ treatment, such as 45% in Tola et al*.* [53]*,* and 55.8% and 58.6% in Oboh et al. [67]; some of them even showing higher prevalence of mutant-type (CV**IET**) than wild-type (CVMNK) haplotypes [68], mainly at sites located in southern regions compared to northern ones [68]. The high prevalence of *pfcrt* 76 T mutation suggest that the CQ pressure has been sustained despite the change in antimalarial drug policy from CQ to ACT, probably as a result of the availability of CQ in medicine stores or as a response to AQ drug pressure [69, 70]. Conversely, this study found a low prevalence of the mutant 76 T *pfcrt* allele (12.12%), likely reflecting the decline in CQ use in the selected population of the study [4, 20].

Mutations on *pfmdr1* gene are involved in resistance to multiple drugs, such as CQ or AQ, and contribute to this resistance along with *pfcrt* SNPs. The Y184F was the *pfmdr1* mutation more frequently found in this study (79.39%), followed by N86Y (2.67%). A previous study revealed the minimal impact of the Y184F mutation on antimalarial drug susceptibilities, contrary to the impact of the N86Y mutation on AQ and CQ resistances [32]. Although the Y184F mutation was highly prevalent in this study, the haplotype comprising *pfmdr1* N86Y and Y184 (the wild-type form of 184 allele), associated with resistance to AQ [33], was not detected. A low prevalence of 86Y mutation have also been reported in other African countries, where arthemeter–lumefantrine is the first-line ACT and where a low percentage of parasite isolates have the lumefantrine-sensitizing N86Y mutation [32]. In contrast, N86Y *pfmdr1*mutation levels increase in countries, where artesunate–amodiaquine is widely use [32], as a response to the drug selective pressure.

As mentioned earlier, combined *pfcrt* and *pfmdr1* haplotypes are associated with resistance to CQ [28, 31] and AQ in *P. falciparum*. AQ is an accompanying drug in artesunate–amodiaquine treatment (a second-line ACT in Nigeria for uncomplicated malaria) and in the SMC strategy (typically with SP plus AQ) [59]. This study did not detect any sample carrying the 86Y/76 T *pfmdr1/pfcrt* mutant haplotype or the YY–CV**IET** haplotype *(pfmdr1* N86Y and wild-type Y184 and *pfcrt* CVIET). Then, it is likely that AQ effectiveness remains acceptably high for the immediate future, as it has been reported elsewhere [33, 69]. Even though the CVIET haplotype of *pfcrt* was present at low prevalence, it was no significant prevalence of the accompanying YY haplotype of *pfmdr1*, which is essential for clinically relevant AQ resistance in Africa [33]*.*

*P. falciparum* genomes carrying simultaneously specific variants of *pfcrt* (CVIET)*, pfmdr1* (YY)*, pfdhps* (GE) and *pfdhfr* (IRN) are likely resistant to both SP and AQ, and could potentially compromise the efficacy of SP plus AQ administered for SMC [33]. However, this study along with previous studies [33] provide evidence that SMC (SP + AQ) is not currently under a serious threat from drug resistant parasites. Nevertheless, continuous surveillance is needed to prevent the emergence of resistance in the future.

The limitations of this study include the lack of correlation studies between the detected *pfk13* mutations with in vivo and in vitro phenotypes, needed to establish the functional role of the detected mutations as markers of artemisinin resistance in Nigeria. Another limitation is the study was conducted at a single site in Nigeria, and it would be very convenient to conduct a study covering multiple regions across the whole country to assess the status of these genes on a larger scale.

## Conclusions

Our analysis of blood samples collected from in children in southwestern Nigeria provides an overview of the molecular resistance markers for *P. falciparum.* Evidence has been found of a validated (C580Y) and a candidate (R515K) mutation in *pfk13* gene, an essential baseline information for tracking and monitoring *P. falciparum* resistance to artemisinin in Nigeria. No samples were found to carry the ‘fully’ or ‘super’ resistant haplotypes, nor *P. falciparum* genomes carrying simultaneously specific variants of *pfcrt* (CV**IET**) and *pfmdr1* (YY) were detected. Therefore, no imminent threat from parasite genomes harbouring multilocus mutations conferring multidrug resistance was identified. However, certain genotypes of concern were observed particularly at the *pfdhps* locus, with the detection of one sample with the 540E *pfdhps* mutation in combination with *pfdhfr* 51I, 59R, and 108N and *pfdhps* 431 V, 436A, A437G and 540E mutations. The study highlights the high frequency of the triple *pfdhfr* mutant (51I, 59R and 108N) and significant variability in *pfdhps* haplotypes, including the emerging **VAG**K**GS** haplotype whose potential role in resistance remains a concern and must still be elucidated.

## Supplementary Information

Additional file 1.

## Data Availability

No datasets were generated or analysed during the current study.

## Abbreviations

ACTs: Artemisinin Combination Therapies
AQ: Amodiaquine
BLAST: Basic Local Alignment Search Tool
COVID-19: Coronavirus disease caused by the SARS-CoV-2 virus
CQ: Chloroquine
DBS: Dried Blood Spot
IPT: Intermittent Preventive Treatment
IPTi: Intermittent Preventive Treatment in infants
IPTp: Intermittent Preventive Treatment during pregnancy
NM–PCR: Nested Multiplex Malaria–PCR
PCR: Polymerase Chain Reaction
*Pfcrt*: Plasmodium falciparum chloroquine resistance transporter gene
*Pfdhfr*: *Plasmodium falciparum* Dihydrofolate reductase gene
*Pfdhps*: *Plasmodium falciparum* Dihydropteroate synthase gene
*Pfk13*: Kelch gene on chromosome 13 of *Plasmodium falciparum*
*Pfmdr*: *Plasmodium falciparum* Multidrug resistance gene
SMC: Seasonal Malaria Chemoprevention
SNP: Single nucleotide polymorphisms
SP: Sulfadoxine/pyrimethamine
RDTs: Rapid Diagnostic Tests
WHO: World Health Organization
