# Supplementary material for: Mutational profile of pfdhfr, pfdhps, pfmdr1, pfcrt and pfk13 genes of P. falciparum associated with resistance to different antimalarial drugs in Osun state, southwestern Nigeria
Source: Trop Med Health. 2025 Apr 8;53:49. doi: 10.1186/s41182-025-00732-6 (PMC11977893; doi:10.1186/s41182-025-00732-6)
Supplement: Supplementary file 1 — Additional file 1. [file 41182_2025_732_MOESM1_ESM.docx]

**Additional Documents**

Additional table 1: Prevalence and percentage of *pfdhfr* and *pfdhps* haplotypes detected in *P. falciparum* isolates (N=266).

| **Haplotypes (N=266)** | | | | |
| --- | --- | --- | --- | --- |
| **Gene** | **Combination of mutated codons** | **Haplotypes** | **Prevalence (N)** | **Percentage** |
| ***pfdhfr*** | Wild type | NCSI | 1 | 0.38% |
|  | 51/59/108 | **IRN**I | 258 | 96.99% |
|  | 51/59/108/164 | **IRNL** | 0 | 0.00% |
|  |  |  |  |  |
| ***pfdhps*** | Wild type | ISAKAA | 0 | 0.00% |
|  | 436 | I**A**AKAA | 3 | 1.13% |
|  | 436 | I**F**AKAA | 1 | 0.38% |
|  | 437 | IS**G**KAA | 50 | 18.80% |
|  | 436/437 | I**FG**KAA | 34 | 12.78% |
|  | 436/437 | I**AG**KAA | 30 | 11.28% |
|  | 436/613 | I**A**AKA**S** | 1 | 0.38% |
|  | 436/437/613 | I**FG**KA**S** | 1 | 0.38% |
|  | 436/437/613 | I**AG**KA**S** | 16 | 6.02% |
|  | 436/437/581 | I**FG**K**G**A | 11 | 4.14% |
|  | 437/613 | IS**G**KA**S** | 2 | 0.75% |
|  | 437/581 | IS**G**K**G**A | 21 | 7.89% |
|  | 437/581/613 | IS**G**K**GS** | 7 | 2.63% |
|  | 431/436/437 | **VAG**KAA | 6 | 2.26% |
|  | 431/436/437/613 | **VAG**KA**s** | 3 | 1.13% |
|  | 431/436/437/581 | **VAG**K**G**A | 12 | 4.51% |
|  | 431/436/437/540 | **VAGE**AA | 1 | 0.38% |
|  | 431/436/437/581/613 | **VAG**K**GS** | 28 | 10.53% |
|  | 431/437/581 | **V**S**G**K**G**A | 3 | 1.13% |
|  | 431/437/613 | **V**S**G**KA**S** | 1 | 0.38% |
|  | 436/437/581 | I**AG**K**G**A | 15 | 5.64% |
|  | 436/581/613 | I**A**AK**GS** | 1 | 0.38% |
|  | 436/581 | I**A**AK**G**A | 3 | 1.13% |
|  | 436/437/581/613 | I**AG**K**GS** | 12 | 4.51% |
|  | 436/437/581/613 | I**FG**K**GS** | 1 | 0.38% |
|  | 431/581/613 | **V**SAK**GS** | 1 | 0.38% |
|  | 431/436/581/613 | **VF**AK**GS** | 1 | 0.38% |
|  | 431/437/581/613 | **V**S**G**K**GS** | 1 | 0.38% |
